# Behavioral Patterns of Sugary Drink Consumption among African American Adolescents: A Pilot and Feasibility Study Using Ecological Momentary Assessment

**DOI:** 10.3390/nu15092171

**Published:** 2023-05-02

**Authors:** Kacey Ferguson, Kathleen Gunthert, Jasmine H. Kaidbey, Meredith Parr, Amanda J. Visek, Jennifer M. Sacheck, Allison C. Sylvetsky

**Affiliations:** 1 Department of Exercise and Nutrition Sciences, Milken Institute School of Public Health, The George Washington University, Washington, DC 20052, USA; 2 Department of Psychology, American University, Washington, DC 20016, USA

**Keywords:** sugar-sweetened beverages, diet, youth, obesity, soda, nutrition

## Abstract

Background: Sugary drinks (SDs) are the predominant contributors to added sugar intake among adolescents, with the highest intakes reported among African American adolescents. The objective of this pilot study was to examine the feasibility of using mobile phone-based ecological momentary assessment (EMA) to investigate, in real time, behavioral patterns of SD consumption among African American adolescents from low-income households. Methods: Adolescents (*n* = 39, ages 12–17) attended a virtual meeting with a trained research assistant, which involved completion of surveys and training on responding to EMA prompts using a mobile phone application. On the seven subsequent days, adolescents were instructed to respond to researcher-initiated prompts three times daily, which queried their SD intake, location, social context, activities, stress, and mood. They were also asked to complete an analogous self-initiated survey each time they consumed SDs. Results: SD consumption was reported on 219 of 582 (38%) researcher-initiated surveys and on 135 self-initiated SD consumption surveys, for a total of 354 instances of SD intake over the 7-day assessment period. The majority (69%) of the surveys were completed while at home. SD consumption was reported on 37%, 35%, and 41% of researcher-initiated surveys completed at their home, at the home of a friend or family member, or while in transit, respectively. Conclusions: These preliminary data indicate that mobile phone-based EMA is feasible for investigating SD intake behaviors among African American youth from low-income households and support the promise of EMA for investigating SD consumption in this population in larger samples of youth.

## 1. Introduction

Approximately one in five youth in the United States (US) has obesity [1]. Obesity in childhood increases the risk of type 2 diabetes, fatty liver, orthopedic disorders [2], depression [3], and low self-esteem [4]. These conditions disproportionately burden African American youth [5], and particularly those from low-income communities. Excess added sugar intake is a key contributor to obesity [6,7], and is an independent risk factor for cardiometabolic disease [8]. Sugary drinks (SDs) are the primary source of added sugar in youths’ diets [9,10]; in addition to being high in sugar and calories, SDs provide little to no nutritional benefit. Consumption of added sugars among youth considerably exceeds recommendations [11,12], with the highest intakes reported among African American youth [13,14].

Disparities in SD intake are explained by an array of multilevel, modifiable factors [13] and reducing SD intake may be particularly challenging for African American youth from low-income households, who are disproportionately exposed to SD advertising [15] and experience greater difficulty with lifestyle modifications due to a myriad of psychosocial, social, and socioeconomic factors [16]. Further, the widespread availability and accessibility of SDs are well-established contributors to continued excess SD consumption. For example, seeing others consuming SDs [17], being in settings where SDs are available or are typically consumed, and/or viewing advertisements for SDs [15] are powerful cues for SD intake [18]. The number and density of these cues one is exposed to strongly impacts dietary choices [19], particularly for discretionary and energy-dense foods and beverages [20].

Based on our prior work [21,22,23], cues associated with adolescents’ SD intake may include situational factors, such as proximity to SD retail [24]; contextual factors, such as stress [25]; interpersonal factors, such as drinking SDs with others; and internal factors, such as physiological (e.g., perceived fatigue) and/or affective (e.g., mood) states [18,22]. However, the roles of these cues in influencing adolescents’ SD intake have not been well-studied to date. This is, in part, because individuals are often not aware of the momentary cues that influence their behaviors, and reliably capturing these cues necessitates real-time assessment tools. 

Identifying and addressing these momentary cues is particularly critical in African American adolescents from low-income families because youth in this sociodemographic subgroup report disproportionately high SD consumption, are unduly burdened by obesity and related comorbidities, and experience multilevel challenges to reducing SD intake [16]. Given the complexity of factors that influence SD intake among African American youth, the use of mobile phone-based ecological momentary assessment (EMA) is an innovative approach to examining SD consumption behaviors in real time; however, it has not been widely utilized in this population subgroup to date. EMA offers advantages over more traditional methods of assessing SD consumption behaviors because it removes the need for retrospective recall of SD intake, and allows for more nuance in understanding complex factors associated with SD consumption. Thus, the purpose of this pilot study was to investigate the feasibility of using real-time, mobile phone-based EMA to examine situational (e.g., location), contextual (e.g., stress), interpersonal (e.g., social setting), and internal (e.g., mood) cues associated with SD intake among African American adolescents from low-income households in Washington DC.

## 2. Materials and Methods

Adolescents were recruited using social media, neighborhood listservs, and a paid community partner from June 2021 to November 2021. They were eligible to participate if they self-identified as African American or Black and met the following criteria: resided in Washington, DC, were between the ages of 12 and 17, reported daily consumption of ≥20 ounces of SDs, such as soda, fruit drinks, fruit juice, and/or sweet tea, and were either eligible for free or reduced-price lunch and/or their parent or guardian reported educational attainment of high school or less. The study protocol (NCR213402) was reviewed and approved by the Institutional Review Board at The George Washington University. The adolescents’ parent or guardian (hereafter parent) provided informed consent electronically and all adolescents provided assent electronically prior to beginning the study procedures.

### 2.1. Study Procedures

Adolescents and their parent were invited to attend a virtual study meeting with a trained research assistant (RA), which was conducted via Zoom^TM^. Data were collected virtually because the study took place during the COVID-19 pandemic, when social distancing measures were in place. After obtaining informed consent and assent electronically using Redcap^TM^, adolescents completed a brief demographic and anthropometric survey. They then responded to the validated BEVQ-15 [26] to assess their usual beverage consumption within the past 30 days and completed four of the five sections of the validated FLEX Diet Questionnaire [27], which queried adolescents’ intake of fruits, vegetables, salty snacks, and sweet snacks over the past week. The fifth category of the FLEX questionnaire, focused on beverage intake, was not administered due to redundancy with the BEVQ-15 [26]. Using the FLEX questionnaire, adolescents indicated how many times in the past week they consumed each item and how much they consumed each time, with response options of a little (1/2 of a standard serving), some (1 serving), or a lot (1.5 servings). Adolescents also completed the Perceived Stress Scale [28], selected items from the validated Physical Activity Questionnaire for Older Children (PAQ-C) [29], and the Positive and Negative Affect Schedule for Children Short Form (PANAS-C short form) [30]. The Perceived Stress Scale is a 14-item instrument designed to measure the degree to which situations in one’s life are appraised as stressful, with higher scores indicative of higher perceived stress [28]. The PANAS-C short form is a self-report questionnaire that uses two 5-item scales (10 items total) to measure positive and negative affect among children. Higher scores on the 5-item positive affect scale indicate higher positive affect and higher scores on the 5-item negative affect scale indicate higher negative affect [30]. All questionnaires were administered electronically via RedCap™. Following questionnaire completion, information on adolescents’ daily schedule (e.g., wake up times, bedtime, timing of school, and extracurricular commitments) was collected by the RA to ensure that the researcher-initiated EMA prompts were administered at times when adolescents could access their mobile phones.

Adolescents were then instructed to download an EMA mobile phone application (mEMA, Ilumivu Inc., Asheville, NC, US), which was used to administer EMA prompts throughout the study. Upon downloading the mobile phone application, each adolescent was assigned a unique mobile code, which was generated automatically. The code allowed the research team to link the data collected through the mobile phone application to each adolescents’ study record in RedCap™. Once the adolescent successfully downloaded the application, they completed a series of test prompts with guidance from the RA to ensure they were able to access the EMA surveys and understood how to complete them. Adolescents were also asked to complete a practice version of each type of EMA assessment (i.e., researcher-initiated, self-initiated, and evening survey) during the Zoom™ meeting to ensure comprehension of the study procedures.

The week-long data collection period began the afternoon or evening of the baseline visit, or the day following the baseline visit, if the baseline visit took place in the evening. Each day for seven days following the baseline visit, adolescents received three researcher-initiated prompts, which were sent at randomly generated times during the morning, afternoon, and evening (except for any time periods that the adolescent indicated they did not have cell phone access). Adolescents were also instructed to complete a self-initiated survey each time they consumed an SD and received text message reminders to initiate a survey when drinking an SD throughout the weeklong data collection period. The researcher-initiated and self-initiated surveys included identical questions about whether the adolescent was drinking an SD, who the adolescent was with, what they were doing, and how they were feeling (i.e., perceived stress and mood). If the adolescent reported SD consumption (irrespective of whether SD consumption was reported on a researcher-initiated survey or whether a self-initiated survey was completed), they were further prompted to answer additional questions regarding the taste, type, and brand of the SD, as well as their physical and emotional feelings in that moment, compared with the hour before drinking the SD. Adolescents were also instructed to complete a brief daily assessment each night before they went to bed (“bedtime survey”), which they were prompted to complete approximately one hour before the time they reported typically going to sleep. The bedtime survey included questions about how stressful they perceived their day to be, how much time they spent doing physical activity, and the total number of SDs they consumed that day.

### 2.2. Data Analyses

Descriptive statistics, including means, standard deviations, and frequencies, as appropriate, were used to summarize adolescents’ responses to the baseline questionnaires. Due to the nested nature of EMA data, we used hierarchical linear modeling (HLM), which employs maximum likelihood estimation and flexibly handles missing data, to calculate frequencies of SD consumption during the 7-day assessment period. We created a two-level model, in which observations within days (level 1) were nested within the participants (level 2). To estimate the proportion of times participants were in each context, we ran a series of unconditional level 1 models where there was a binomial (0 or 1) outcome for each context (e.g., 0 for “not home” and 1 for “at home”). We also used multilevel logistic regression to estimate the change in likelihood of SD consumption in specific contexts as an exploratory analysis due to the small sample size and pilot nature of the study.

## 3. Results

Forty-two adolescents enrolled in the study. However, one adolescent was unresponsive following enrollment, and two additional adolescents were excluded from the analysis due to non-compliance with the study procedures. Thirty-nine adolescents completed the full study protocol. As shown in Table 1, the sample comprised 41% boys and 59% girls, who were on average 14 years old. Most were from households with an educational attainment of high school or less (63%). Over half of the adolescents were overweight or had obesity, based on BMI percentile, which was calculated using their self-reported height and weight. Habitual SD intake in the sample was high, with most adolescents reporting consumption of more than two servings of SDs daily and over one-third reporting usual consumption of four or more servings of SDs per day.

As shown in Table 2, a total of 833 researcher-initiated surveys were sent over the course of the study, 582 (median 2 per participant per day) of which were completed (70%). Adolescents reported 219 instances (38%) of consuming SDs when responding to a researcher-initiated survey and 135 instances of SD consumption (median of 0 per participant per day) in self-initiated surveys, resulting in 354 SD consumption instances reported collectively throughout the study. Over half of the participants completed at least two of the three researcher-initiated surveys administered each day; however, the average completion of self-initiated SD consumption surveys was fewer than one per day.

As shown in Table 3, 68% of random, researcher-initiated surveys were completed while adolescents were at home. On random, researcher-initiated surveys completed at home, adolescents indicated SD intake 37% percent of the time. SD intake was also commonly reported (38%) on random, researcher-initiated surveys completed when at the home of friends or family members, in transit (41%), outside (26%), and at extracurricular activities (25%). While only a small number of surveys were completed while adolescents were at school (attributable to reduced access to mobile phones during the school day and enrollment taking place primarily during summer months), SD intake was reported on 19% of random, researcher-initiated surveys (*n* = 37) completed at school.

In terms of social context, most of the random, researcher-initiated surveys were completed while adolescents were with family and/or friends (71%), while approximately 29% were completed while the adolescent was alone (Table 3). Adolescents reported that they were using technology on approximately 39% of the random, researcher-initiated surveys.

Adolescents’ perceived stress and positive and negative affect were similar regardless of whether SD consumption was reported. However, on instances when an SD was consumed, participants reported higher positive affect after consuming the SD compared with the hour before SD consumption (16.2 ± 6.8 versus 15.0 ± 7.3 after compared with before SD consumption, *p* = 0.002). In contrast, negative affect scores were similar after versus one hour prior to consuming an SD (5.7 ± 1.9 versus 5.8 ± 2.3 after compared with before SD consumption, not statistically significant).

## 4. Discussion

To our knowledge, this is the first study to use mobile phone-based EMA to evaluate, in real time, situational (e.g., location), contextual (e.g., stress), interpersonal (e.g., social setting), and internal (e.g., mood) cues associated with SD consumption specifically among African American adolescents from low-income families. Importantly, studies of dietary behaviors typically rely on retrospective surveys, where participants are asked to recall their behavior over the past day(s), week(s), or month(s). These self-report retrospective approaches have questionable reliability and use of EMA allowed us to capture adolescents’ SD intake in real time by assessing their feelings, experiences, and behaviors within their natural environment, and in the context of their usual SD consumption.

Considering that SD consumption was reported on more than one in three (38%) of the researcher-initiated surveys sent at random times, in addition to on self-initiated SD consumption surveys, the present findings call attention to the strikingly high frequency of SD consumption in this population subgroup. It is noteworthy that reported consumption of at least 20 ounces of SDs daily was an inclusion criterion and the findings therefore may not be generalizable to low-income, African American adolescents more broadly. However, within this sample, it is notable that most participants reported SD consumption that far exceeded the inclusion criterion of habitually consuming at least 20 ounces of SDs per day.

A notable finding of this analysis was that most of the surveys were completed while adolescents were at home. This emphasizes the crucial role of the home environment in SD consumption behaviors [18,23,31,32,33,34,35] and calls attention to the importance of interventions (e.g., education for parents and point-of-purchase interventions to discourage SD purchases) to restrict access to SDs in the home and support youth in establishing healthy habits from an early age. A previous study demonstrated that home environmental factors were mediated by parental rules and habit strength, and showed that greater exposure to SDs at home encouraged SD consumption [34]. Adolescence marks a period of increased autonomy and independence over dietary behaviors [36]; therefore, it is particularly imperative for parents to limit the availability of SDs in the home and play an active role (e.g., modeling healthy behaviors) in reducing their consumption.

It is also noteworthy that adolescents reported being with family on over 50% of researcher-initiated surveys sent at random times, which emphasizes the need to target the whole family (and not just the adolescent) in efforts to lower SD consumption. This is not surprising as positive associations between parents’ and adolescents’ intakes of SDs are well-documented [23,31,32,34,37] and prior interventions to reduce SD intake among youth have shown promising benefits for SD reduction when parents are engaged [38]. It is therefore critical for parents to model healthy beverage consumption behaviors by modifying their own SD consumption, as well as actively participating in limiting their child’s SD access and discouraging their child from drinking SDs.

While the number of surveys completed in the restaurant or store setting was small (*n* = 4), adolescents indicated SD intake on 75% of the researcher-initiated surveys completed in this setting. In the United States from 2005 to 2006, approximately one-third of SDs consumed were obtained at restaurants and fast-food retail outside of the home [39], and other research shows that SD consumption at restaurants has increased over time [33]. This is important to consider with regard to the disproportionate abundance of unhealthy food retail outlets (e.g., fast food venues and convenience stores) compared with supermarkets in communities with a greater proportion of residents from low-income and/or historically marginalized backgrounds [40,41,42,43]. This widespread access to SDs represents a key barrier to healthy beverage choices [40,41,42,43] and may also explain the frequently reported SD intake indicated on surveys completed while adolescents were outside or in transit.

Use of technology, such as watching television or using a computer or social media, was also commonly reported on researcher-initiated surveys. Widespread use of technology among adolescents in the present study suggests that using technology may provide an opportunity to educate adolescents about SD intake and discourage SD intake and/or encourage healthier beverage choices. For example, showing parents counter-marketing videos addressing misperceptions about the healthfulness of SDs has been shown to be promising for reducing SD intake among young children [44]. A similar approach could also be useful with adolescents and could be administered using social media.

Interestingly, on instances when adolescents reported consuming a SD, they reported higher positive affect while consuming the SD, compared to the hour prior to consumption. While this finding should be interpreted with caution due to the small sample size, this is consistent with our prior qualitative findings demonstrating that youth experience positive emotions when drinking SDs [22]. Actual, or perceived, mood enhancing effects of SDs may represent an important and overlooked contributor to excess SD consumption among youth [45]; and, negative emotions resulting from restriction of SDs among children have also been previously described [23,46]. However, aversive responses to SD consumption, such as aggressiveness and irritability, nervousness, and anxiety-induced sleep disturbances have also been reported among children [47,48,49], in addition to our previous findings that three days of SD cessation resulted in reported improvements in children’s affect [50]. Nonetheless, emotional factors associated with SD consumption have been understudied to date, and effects of SD intake on mood and affect among youth is an important area for future research and is well-suited to assessment using real-time EMA.

### Strengths and Limitations

A key strength of our pilot study was the ability to assess situational, contextual, interpersonal, and internal cues associated with adolescents’ SD intake in real time, using EMA to examine SD consumption behaviors both within and between subjects. This allowed us to produce the rich, ecologically valid data that are needed to capture linkages between cues and SD consumption behaviors. The present study also specifically focused on SD consumption behaviors among African American adolescents from low-income households in a historically marginalized and underserved community in Washington, DC, where the burden of diet-related cardiometabolic disease is disproportionately high [16,51]. Furthermore, adherence to completion of the researcher-initiated surveys was high, which suggests that use of mobile phone-based EMA is feasible in this population and may hold promise for delivering intervention content to reduce SD intake in this and similar populations in future studies.

The main weakness of our analysis was the small sample size due to the pilot and feasibility nature of the study, which did not allow for robust statistical comparisons. Furthermore, in contrast to high adherence to completion of researcher-initiated surveys, adolescents completed only 0.5 self-initiated SD consumption surveys per day on average. Given the high frequency of SD intake reported at baseline (on average, more than two servings/day), the low completion of self-initiated SD intake surveys likely reflects non-compliance with instructions to complete a self-administered survey each time adolescents consumed SDs and/or may indicate that adolescents changed their behavior during the study (i.e., they consumed SDs less frequently than usual). In addition, data collection took place both during the summer months and the school year when adolescents likely had different schedules and therefore may have been differentially exposed to key SD consumption cues. Further, many adolescents were unable to use their mobile phone while at school, which limited the time windows that EMA prompts could be administered and/or completed, precluding comparison with adolescents who completed the study during the summer.

## 5. Conclusions

Our findings support the feasibility of using mobile phone-based, real-time EMA prompts, sent several times a day for one week, to investigate cues associated with SD consumption among youth from a historically marginalized and underserved community. Our study lays the groundwork for larger, sufficiently powered studies of SD consumption behaviors using this approach. Furthermore, considering that completion of self-initiated SD consumption surveys was relatively low in our sample, these findings suggest that increasing the number of random surveys may provide an opportunity to obtain more detailed, time-varying information, while eliminating the need to rely on completion of self-initiated surveys. Finally, a precision nutrition approach that leverages mobile phone-based EMA and targets multiple settings and contexts where SDs are consumed may offer a promising strategy for future interventions to lower SD intake among underserved youth. EMA studies of SD consumption behaviors in larger samples of African American adolescents are needed to guide the development of tailored interventions designed to modulate adolescents’ responses to cues for SD intake, which will increase adherence to ongoing efforts focused on reducing SD consumption.

## Figures and Tables

**Table 1:** Characteristics of the study participants, *n* = 39.

	*n* (%) ^1^
**Sex**	
Male	16 (41.0)
Female	23 (59.0)
**Age, years** (mean ± SD)	14.0 ± 1.8
**Race**	
African American or Black	34 (87.2)
Mixed race, including African American or Black	5 (12.8)
**Ethnicity**	
Hispanic or Latino	2 (5.1)
Not Hispanic or Latino	37 (94.9)
**Highest level of education in household**	
Less than high school	18 (46.2)
Completed high school	7 (18.0)
Some college or vocational training	6 (15.4)
Completed college or university or higher	9 (23.1)
**BMI Percentile** (mean ± SD)	77.4 ± 25.5
**Weight Status** ^2^	
Healthy weight	18 (46.2)
Overweight	6 (15.4)
Obesity	15 (38.5)
**Daily SD consumption, 12-ounce servings** ^3^	
<1	4 (10.3)
1–2	7 (17.9)
>2–4	13 (33.3)
>4	15 (38.5)
**Dietary intake, servings per day** ^4^	
Fruit intake (servings/day)	1.6 ± 1.6
Vegetable intake (servings/day)	1.3 ± 1.5
Salty snack intake (servings/day)	1.5 ± 1.2
Sweet snack intake (servings/day)	1.2 ± 1.0
**Physical activity, days per week meeting physical activity recommendation** ^5^	4.1 ± 2.1
**Positive affect** ^6^ (max score = 25)	18.0 ± 4.5
**Negative affect** ^7^ (max score = 25)	6.6 ± 2.1
**Perceived stress** ^8^ (max score = 56)	23.1 ± 6.6

Abbreviations: BMI = body mass index; SD = sugary drink. Bolded text represents response categories, while unbolded text represents sub-categories. ^1^ Some percentages do not sum to 100% due to rounding. ^2^ Using standard CDC cut-offs for BMI-for-age: healthy weight = 5th to <85th percentile; overweight = 85th to <95th percentile; obesity = ≥95th percentile. ^3^ Four participants reported habitual consumption of less than 12 ounces of SDs per day using the beverage questionnaire administered during the baseline visit, despite reporting that they consumed ≥12 ounces of SDs per day when screened for study eligibility. ^4^ Assessed using the validated FLEX diet questionnaire. ^5^ Assessed using the following item from the validated PAQ-Q: “During the past 7 days, on how many days were you physically active for a total of at least 60 min per day?”. ^6^ Assessed using the following PANAS-C short form items: joyful, cheerful, happy, lively, and proud. ^7^ Assessed using the following PANAS-C items: miserable, mad, afraid, scared, and sad. ^8^ Assessed using the validated perceived stress scale.

**Table 2:** Adolescents’ responses to ecological momentary assessment prompts, by prompt type and SD consumption.

	*n*, %
Random, researcher-initiated prompts sent	833 (86.1)
Random, researcher-initiated prompts completed	582 (69.9)
Random researcher-initiated prompts with SD consumption reported	219 (37.6)
Self-initiated surveys completed	135 (13.9)
Total SD intake occasions	354 (49.4)
	**Median (range)**
Random, researcher-initiated prompts completed per participant per day	2 (0–3)
Self-initiated surveys recorded per participant per day	0 (0–4)

**Table 3:** Adolescents’ location, social context, and technology use on random, researcher-initiated surveys, by reported SD intake.

	Number of Surveys with Reported SD Intake (%)
**Where are you right now?**	
At home (*n* = 382)	143 (37.4)
At an extracurricular (*n* = 16)	4 (25.0)
At the home of a friend or family member (*n* = 16)	6 (37.5)
At school (*n* = 37)	7 (18.9)
Transit (*n* = 70)	29 (41.4)
Outside (*n* = 38)	10 (26.3)
Other (*n* = 4) ^1^	3 (75.0)
**Who are you with right now?**	
By myself (*n* = 170)	62 (36.5)
With family member(s) (*n* = 300)	117 (39.0)
With friends/peers (*n* = 62)	26 (41.9)
With family and friends (*n* = 47)	13 (27.7)
**What are you doing right now?**	
Using technology (*n* = 224)	86 (38.4)
Not using technology (*n* = 359)	133 (37.0)

Bolded text represents survey questions, while unbolded text represents survey response options. ^1^ Participants who selected other were asked to describe their location. Of the 4 instances where “other” was selected, 3 instances involved being at a restaurant and 1 involved being at a store.

## Data Availability

Data will be made available upon reasonable request to the corresponding author.
